# Structural insights into stressosome assembly

**DOI:** 10.1107/S205225251900945X

**Published:** 2019-08-21

**Authors:** Eunju Kwon, Deepak Pathak, Han-ul Kim, Pawan Dahal, Sung Chul Ha, Seung Sik Lee, Hyeongseop Jeong, Dooil Jeoung, Hyeun Wook Chang, Hyun Suk Jung, Dong Young Kim

**Affiliations:** a College of Pharmacy, Yeungnam University, Gyeongsan, Gyeongbuk 38541, Republic of Korea; b Department of Biochemistry, College of Natural Sciences, Kangwon National University, Chuncheon, Gangwon 24341, Republic of Korea; c Pohang Accelerator Laboratory (PAL), Pohang University of Science and Technology, Pohang, Gyeongbuk 37673, Republic of Korea; d Advanced Radiation Technology Institute, Korea Atomic Energy Research Institute, Jeongeup 56212, Republic of Korea; e Department of Radiation Science and Technology, University of Science and Technology, Daejeon 34113, Republic of Korea; f Center for Electron Microscopy Research, Korea Basic Science Institute, Ochang 28119, Republic of Korea

**Keywords:** stressosome, pseudo-icosahedron, STAS domain, crystal structure, cryo-electron microscopy, X-ray crystallography, single-particle cryo-EM

## Abstract

The stressosome transduces environmental stress signals to SigB to upregulate SigB-dependent transcription, which is required for bacterial viability. The stressosome core is composed of RsbS and at least one of the RsbR paralogs. A previous cryo-electron microscopy (cryo-EM) structure of the RsbRA–RsbS complex determined under a *D*2 symmetry restraint showed that the stressosome core forms a pseudo-icosahedron consisting of 60 STAS domains of RsbRA and RsbS. However, it is still unclear how RsbS and one of the RsbR paralogs assemble into the stressosome. Here, an assembly model of the stressosome is presented based on the crystal structure of the RsbS icosahedron and cryo-EM structures of the RsbRA–RsbS complex determined under diverse symmetry restraints (nonsymmetric *C*1, dihedral *D*2 and icosahedral *I* envelopes). 60 monomers of the crystal structure of RsbS fitted well into the *I*-restrained cryo-EM structure determined at 4.1 Å resolution, even though the STAS domains in the *I* envelope were averaged. This indicates that RsbS and RsbRA share a highly conserved STAS fold. 22 protrusions observed in the *C*1 envelope, corresponding to dimers of the RsbRA N-domain, allowed the STAS domains of RsbRA and RsbS to be distinguished in the stressosome core. Based on these, the model of the stressosome core was reconstructed. The mutation of RsbRA residues at the binding interface in the model (R189A/Q191A) significantly reduced the interaction between RsbRA and RsbS. These results suggest that nonconserved residues in the conserved STAS folds between RsbS and RsbR paralogs determine stressosome assembly.

## Abstract 2

Stressosome assembly mediated by the STAS domain was analyzed based on the crystal structure of RsbS and the cryo-EM structure of the RsbS–RsbRA complex.

## 1. Introduction   

Sigma/antisigma signaling pathways are widely used in bacteria to adapt to environmental conditions altered by diverse stresses (Hughes & Mathee, 1998 ▸; Raivio & Silhavy, 2001 ▸; Gruber & Gross, 2003 ▸). In the signaling pathways, stress-sensing proteins are activated in response to abnormal conditions and the signal is transduced to activate the transcription of a target regulon, resulting in adaptation to the altered environment. For example, *Escherichia coli* DegS recognizes the C-termini of unfolded outer membrane proteins (OMPs; Walsh *et al.*, 2003 ▸; Wilken *et al.*, 2004 ▸) and initiates a proteolytic cascade for the activation of SigE (Alba *et al.*, 2002 ▸; Kanehara *et al.*, 2002 ▸; Flynn *et al.*, 2004 ▸). The activated SigE induces the overexpression of heat-shock proteins to confer *E. coli* with resistance against stresses that cause the unfolding of OMP proteins (Rhodius *et al.*, 2006 ▸).

SigB from *Bacillus subtilis* induces the transcription of a SigB-dependent regulon in response to diverse environmental stresses, including heat, ethanol and osmotic shock (Voelker *et al.*, 1995 ▸; Boylan *et al.*, 1993 ▸; Benson & Haldenwang, 1993 ▸). The stressosome, which is composed of RsbR, RsbS and RsbT, is known to mediate stress signaling for SigB activation (Chen *et al.*, 2003 ▸). The stress signal is transferred downstream in the following order: RsbR/S, RsbT, RsbU, RsbV, RsbW and SigB. Initially, the kinase RsbT phosphorylates Ser59 of RsbS in response to stress (Kang *et al.*, 1996 ▸; Yang *et al.*, 1996 ▸). This phosphorylation causes the release of RsbT from the stressosome, followed by the activation of the phosphatase RsbU through direct interaction with RsbT (Yang *et al.*, 1996 ▸; Delumeau *et al.*, 2004 ▸). Next, RsbV dephosphorylated by RsbU releases SigB by hijacking the antisigma factor RsbW from the SigB–RsbW complex. Finally, SigB, as a subunit of the RNA polymerase holoenzyme, induces the transcription of the target regulon required for the stress adaptation (Hecker *et al.*, 2007 ▸; Völker *et al.*, 1994 ▸; Yang *et al.*, 1996 ▸). The phosphorylated stressosome is returned to the pre-stress form by the phosphatase RsbX (Yang *et al.*, 1996 ▸; Chen *et al.*, 2004 ▸).

The stressosome is sparsely found in diverse bacterial species (Jia *et al.*, 2016 ▸; Pané-Farré *et al.*, 2005 ▸). The most studied stressosome, that from *B. subtilis*, forms a large protein complex that comprises RsbR, RsbS and RsbT (Akbar *et al.*, 1997 ▸; Chen *et al.*, 2003 ▸; Delumeau *et al.*, 2006 ▸; Marles-Wright *et al.*, 2008 ▸). Five RsbR family proteins (RsbRA, RsbRB, RsbRC, RsbRD and YtvA) have been identified in *B. subtilis*, each of which recognizes different environmental changes (Akbar *et al.*, 2001 ▸; Kim *et al.*, 2004 ▸; Avila-Pérez *et al.*, 2006 ▸; Gaidenko *et al.*, 2006 ▸). RsbR family proteins consist of an N-terminal nonheme globin domain and a C-terminal STAS (sulfate transporter/antisigma factor antagonist) domain, except for YtvA, which contains an N-terminal LOV (light–oxygen–voltage-sensing) domain (Murray *et al.*, 2005 ▸; Marles-Wright *et al.*, 2008 ▸). Unlike the RsbR proteins, RsbS only contains an STAS domain. RsbS forms a large complex in the presence of at least one of the RsbR paralogs (Kim *et al.*, 2004 ▸; Delumeau *et al.*, 2006 ▸). The cryo-electron microscopy (cryo-EM) structure of the RsbRA–RsbS complex determined under a *D*2 symmetric restraint suggested that the STAS domains of 40 RsbRA and 20 RsbS molecules form a stressosome core with a truncated icosahedral shape (Marles-Wright *et al.*, 2008 ▸; Marles-Wright & Lewis, 2010 ▸) and dimeric N-terminal domains of RsbRA project outwards from the stressosome core. Although the assembly mode of RsbT has not yet been determined clearly in the cryo-EM structure of the stressosome (the RsbRA–RsbS–RsbT complex), RsbT seems to interact with the outer surface of the stressosome core, because it is dissociated from the stressosome core and interacts with RsbU to transfer the activation signal.

Here, we report an assembly model of the stressosome core based on the crystal structure of *B. subtilis* RsbS and cryo-EM structures of the RsbRA–RsbS complex. Unlike the structures of known STAS proteins, the crystal structure of RsbS showed an icosahedral assembly of 60 RsbS monomers and fitted well into the cryo-EM structure of the stressosome core determined at 4.1 Å resolution with an icosahedral symmetric restraint. The cryo-EM structure of RsbRA–RsbS determined at 9.1 Å resolution without symmetric restraints allowed the positioning of the STAS domain of RsbRA in the icosahedral stressosome core. Our model of the stressosome core shows how RsbRA and RsbS assemble into a pseudo-icosahedral structure.

## 2. Methods   

### 2.1. Plasmid preparation, expression and purification   

The genes encoding full-length RsbS (residues 1–121) and RsbRA (residues 1–274) were amplified using the polymerase chain reaction (PCR) from the genomic DNA of *B. subtilis* strain 168 (ATCC No. 23857). The *rsbS* and *rsbRA* genes were inserted into pET-DUET1 and pET-22b vectors (Novagen; Merck Millipore, Billerica, Massachusetts, USA) to express 6×His-thioredoxin-RsbS and native RsbRA, respectively.

*E. coli* strain BL21 Star (DE3) cells were then transformed using each plasmid (Invitrogen; Thermo Fisher Scientific, Waltham, Massachusetts, USA) and the cells were grown in LB medium. The overexpression of 6×His-thioredoxin-RsbS and RsbRA was induced by the addition of 0.4 m*M* isopropyl β-d-1-thiogalactopyranoside at 20°C. After overnight culture, the cells were harvested by centrifugation and resuspended in buffer *A* (20 m*M* Tris pH 8.5, 0.3 *M* NaCl, 5% glycerol). The resuspended cells were disrupted by sonication and then clarified by centrifugation at 20 000*g* for 30 min after incubation with DNase I (Roche, Mannheim, Germany) and RNaseA (Roche) at a concentration of 10 µg ml^−1^.

RsbS was purified by immobilized metal-affinity chromatography (IMAC) and size-exclusion chromatography (SEC). The clarified cell lysate was loaded onto a HiTrap IMAC column (GE Healthcare Life Sciences, Logan, Utah, USA) and RsbS was eluted with a 0.05–0.5 *M* imidazole gradient. The protein purified by IMAC was treated with TEV protease to remove the N-terminal 6×His-thioredoxin tag from RsbS. After RsbS and the 6×His-thioredoxin tag had been completely separated, the protein solution was dialyzed in buffer *A* and passed through nickel-charged resin (Qiagen, Hilden, Germany) to remove 6×His-thioredoxin. RsbS was further purified on a Superdex 75 size-exclusion column (GE Healthcare Life Sciences) equilibrated with buffer *A*. The purified RsbS was concentrated to 10 mg ml^−1^ and was estimated to be >95% pure by SDS–PAGE (sodium dodecyl sulfate–polyacrylamide gel electrophoresis). Selenomethionine-labeled RsbS was expressed in *E. coli* strain BL21 Star cells grown in minimal medium containing selenomethionine (TCI Chemicals, Tokyo, Japan) and was purified using the same procedures as used for native RsbS and described above.

The RsbS–RsbRA complex was purified using similar procedures to those used for RsbS. *E. coli* cells in which 6×His-thioredoxin-RsbS and RsbRA had separately been expressed were mixed with an excess of 6×His-thioredoxin-RsbS. The complex was purified by IMAC and treated with TEV protease to separate 6×His-thioredoxin from RsbS. The 6×His-thioredoxin was removed by passage through nickel-charged resin and excess RsbS was removed using a Superdex 200 size-exclusion column (GE Healthcare Life Sciences) equilibrated with buffer *A*.

### 2.2. Crystallization, data collection and structure determination   

RsbS crystals suitable for X-ray data collection were grown in a micro-batch plate at 20°C. The crystallization drop was prepared by mixing 1 µl protein solution (10 mg ml^−1^) and 1 µl crystallization solution (35% MPD, 100 m*M* MES/sodium hydroxide pH 6.0, 200 m*M* lithium sulfate) under a layer of Al’s oil (Hampton Research, Aliso Viejo, California, USA). For cryoprotection of the crystal, 0.25 µl 100% glycerol was directly added to the crystallization drop, allowing the slow diffusion and equilibration of glycerol. The crystal was then picked up with a cryo-loop (Hampton Research) and flash-cooled in a cold nitrogen stream. The diffraction data were collected on BL7A at Pohang Light Source (PLS), Republic of Korea (Park *et al.*, 2017 ▸) and were indexed, integrated and scaled using *HKL*-2000 (Otwinowski & Minor, 1997 ▸). The diffraction data for the selenomethionine derivative were collected at the peak wavelength of selenium.

The crystal structure of RsbS was determined using the SAD method. The initial experimental map was calculated in *PHENIX* (Adams *et al.*, 2010 ▸) and manual model building was performed using *Coot* (Emsley *et al.*, 2010 ▸). Cycles of refinement and model rebuilding were performed at 3.2 Å resolution using *phenix.refine* (Adams *et al.*, 2010 ▸; Afonine *et al.*, 2012 ▸) and *Coot* (Emsley *et al.*, 2010 ▸). The structure of native RsbS was refined and rebuilt at 3.1 Å resolution after molecular replacement using the model of the seleno­methio­nine derivative as a template. The final structure of native RsbS was refined with *R* and *R*_free_ values of 21.0% and 24.1%, respectively. No residues fell in the disallowed region of the Ramachandran plot, except for Leu23 in chain *E*. The data-collection and refinement statistics for the crystal structure of RsbS are summarized in Table 1 ▸. The figures were drawn using *PyMOL* (v.1.8; Schrödinger) and *ALSCRIPT* (Barton, 1993 ▸). The surface area, protein–protein interaction and structural alignment were analyzed using *PISA* (Krissinel & Henrick, 2007 ▸), *DIMPLOT* (Laskowski & Swindells, 2011 ▸) and the *DALI* server (Holm & Rosenström, 2010 ▸), respectively. The final coordinates and structure factors of RsbS have been deposited in the Protein Data Bank as PDB entry 6jhk.

### 2.3. Transmission electron microscopy and single-particle image processing   

The RsbRA–RsbS complex was diluted with buffer *B* (20 m*M* HEPES pH 7.5, 50 m*M* NaCl, 0.2 m*M* TCEP, 5% glycerol) to a final concentration of 0.5 mg ml^−1^. To obtain negatively stained EM images, the protein sample was applied onto carbon-coated grids that had been glow-discharged (Harrick Plasma, Ithaca, New York, USA) for 3 min in air. The grid was then negatively stained using 1% uranyl acetate (Chung *et al.*, 2018 ▸; Umeki *et al.*, 2011 ▸). Specimens on the grid were observed with a Tecnai 10 transmission electron microscope (TEM) operated at 100 kV (FEI, Hillsboro, Oregon, USA) and the EM images were recorded using an UltraScan 1000 CCD camera (Gatan, Pleasanton, California, USA) at a nominal magnification of 34 000×.

For cryo-EM, frozen-hydrated specimens were prepared on glow-discharged Quantifoil R 1.2/1.3 holey carbon EM grids (Quantifoil, Grosslöbichau, Germany) using a Vitrobot Mark IV (FEI; 7 s blotting time and 100% humidity at 4°C). Automated data collection was performed using the FEI *EPU* software with a Titan Krios G2 transmission electron microscope (FEI) that was operated at 300 kV and was equipped with a Falcon III direct electron detector (DED) (instrumentation installed at Korea Basic Science Institute, Ochang, Republic of Korea). Each 30-frame movie was recorded with an exposure time of 1.8 s, yielding a total dose of 30 e^−^ Å^−2^ per movie and a defocus range from 1.5 to 3.0 µm. Movie motion was corrected using *MotionCor*2 (Zheng *et al.*, 2017 ▸). After estimating the contrast transfer function (CTF) using *Gctf* (Zhang, 2016 ▸), 948 micrographs with values of better than 5 Å resolution and 3 µm defocus were selected for further processing. Particles were picked with *Gautomatch* (http://www.mrc-lmb.cam.ac.uk/kzhang/Gautomatch/). A total of 54 798 particles were extracted using *RELION*-2.1 (Kimanius *et al.*, 2016 ▸). After performing 12 rounds of 2D classification in *RELION*-2.1 (Heymann & Belnap, 2007 ▸), the best-looking 2D class averages were selected as judged by visual inspection. Next, initial reference models with dihedral *D*2, icosahedral *I* or nonsymmetric *C*1 restraints were built from 3240 particles of the best 2D classes in *RELION*-2.1 (Supplementary Fig. S1). 42 623 particles were then selected from the best matched classes with a reference model and were subjected to 3D auto-refinement with *D*2, *I* or *C*1 restraints in *RELION*-2.1. For 3D reconstruction with each symmetry restraint, the particles were polished using initial envelope models calculated with corresponding symmetry restraints and 3D auto-refinement was performed using the polished particles. The final envelope model was refined with a soft-edged mask and was sharpened with a *B* factor. The *B* factors used for the *D*2-, *I*- and *C*1-restrained maps were −303.954, −255.194 and −846.868 Å^2^, respectively.

The resolution of the final cryo-EM structure was estimated by Fourier shell correlation (FSC) between the two halves of the data set using the FSC validation server of the Electron Microscopy Data Bank (EMDB). The local resolution was calculated with the *blocres* program in the *Bsoft* package (Heymann & Belnap, 2007 ▸). The procedure for cryo-EM processing is described in Supplementary Fig. S1. The FSC curves and local resolution are shown in Supplementary Fig. S2. The data-collection and refinement statistics for the cryo-EM structure are summarized in Table 2 ▸. The final cryo-EM envelopes were deposited in the EMDB (EMDB IDs EMD-9923 for the *C*1-refined map, EMD-9924 for the *I*-refined map and EMD-9953 for the *D*2-refined map). *UCSF Chimera* was used to superpose model structures into cryo-EM envelopes and to perform the energy minimization of model structures (Pettersen *et al.*, 2004 ▸). Energy minimization was performed by executing 100 steps of the steepest-descent method with 0.02 Å step size and ten steps of the conjugate-gradient method with 0.02 Å step size.

### 2.4. Peptide mass fingerprinting (PMF)   

To identify the phosphorylation site on RsbS, purified RsbS was digested with trypsin (Promega, Madison, Wisconsin, USA), mixed with α-cyano-4-hydroxycinnamic acid in 50% acetonitrile/0.1% trifluoroacetic acid and subjected to MALDI-TOF analysis (Microflex LRF 20; Bruker Daltonics, Bremen, Germany). Spectra were collected with 300 shots per spectrum over the *m*/*z* range 600–3000 and were calibrated by two-point internal calibration using trypsin autodigestion peaks (*m*/*z* 842.5099 and 2211.1046). The peak list was generated using *FlexAnalysis* 3.0 (Bruker Daltonics). The spectra were analyzed using the *Mascot* software (Matrix Science, London, England). The mass tolerance for the database search was ±0.1 Da.

### 2.5. SEC-MALS   

The protein size was measured by injecting 200 µl 1 mg ml^−1^ RsbS into a Superdex 200 analytical column with an ÄKTApurifier FPLC (GE Healthcare) and the elution products were analyzed using inline miniDAWN TREOS II MALS and Optilab rEX differential refractive-index detectors (Wyatt Technology, Santa Barbara, California, USA). The data were analyzed using the *ASTRA* 6 software package (Wyatt Technology).

### 2.6. GST pulldown   

The genes for RsbS and RsbRA were inserted into pETDuet1 vector with DNA fragments encoding His_6×_-GST and pET-28b vector (Novagen) to express His-GST-RsbS and RsbRA, respectively. Point mutations were introduced into RsbRA by the site-directed mutagenesis method. All primers for the mutagenesis are listed in Supplementary Table S1. His-GST-RsbS and RsbRA mutants were separately expressed in *E. coli* BL21 Star (DE3) cells. The cells were collected by centrifugation and resuspended in buffer *C* (20 m*M* HEPES pH 7.5, 0.2 *M* NaCl, 5% glycerol). The cell lysates were prepared by sonication followed by centrifugation at 20 000*g*. Clarified cell lysates expressing RsbS were mixed with RsbRA and its mutants. They were then loaded onto GST Sepharose (GE Healthcare Life Sciences) and washed with buffer *C*. The proteins bound to the resin were eluted using 2× sample buffer for SDS–PAGE (125 m*M* Tris–HCl pH 6.8, 4% SDS, 20% glycerol, 0.2 *M* DTT, 0.2% bromophenol blue).

## 3. Results and discussion   

### 3.1. Structure determination of *B. subtilis* RsbS   

**Figure 1:**
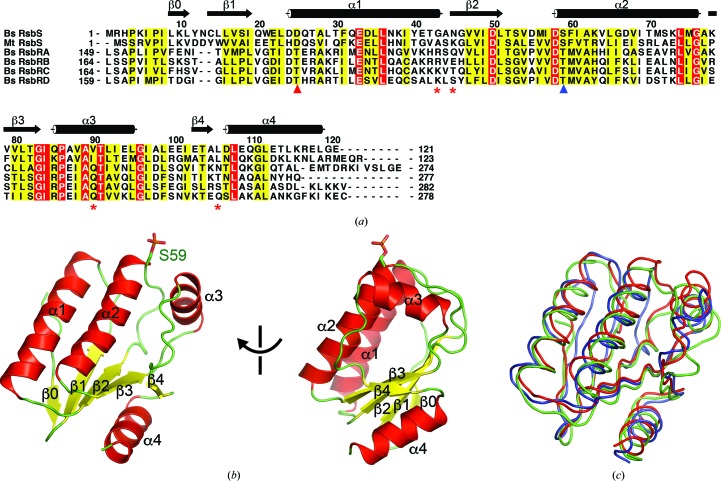
The structure of the RsbS monomer. (*a*) Sequence alignment of STAS proteins. The sequences of *B. subtilis* (Bs) RsbS, *M. thermoacetica* (Mt) RsbS and Bs RsbR proteins (RsbRA, RsbRB, RsbRC and RsbRD) were aligned. Identical and homologous residues are boxed in red and yellow, respectively. The secondary structure of Bs RsbS is displayed using cylinders for α-helices and arrows for β-strands. The phosphorylation sites on RsbS and RsbR, which are crucial for the regulation of SigB activation signaling, are marked with blue and red triangles, respectively. (*b*) The structure of the RsbS monomer. The ribbon models are shown in two different orientations. α-Helices, β-strands and loops are colored red, yellow and green, respectively. The secondary structures are labeled using the same scheme as in (*a*). The phosphorylated Ser59 is shown as a stick model. (*c*) Structural comparison of STAS proteins. The structures of mtSTAS (PDB entry 3ztb; Quin *et al.*, 2012 ▸) and gsSpoIIAB (PDB entry 1til; Masuda *et al.*, 2004 ▸) were superimposed onto RsbS. C^α^ trace models were drawn in the same orientation as in the left panel of (*b*). Red, green and blue represent RsbS, mtSTAS and gsSpoIIAB, respectively.

RsbS was expressed in *E. coli* and purified using nickel-affinity and size-exclusion chromatography. Cubic crystals belonging to space group *I*23 were grown under diverse precipitant conditions, such as NaCl, ethanol and polyethylene glycol 8000. Diffraction data were collected to 3.1 Å resolution. Although RsbS shares 37.9% sequence identity with the *Moorella thermoacetica* STAS (mtSTAS) protein [Fig. 1 ▸(*a*)], the phases could not be obtained by molecular replacement using the structure of mtSTAS, probably because of the large number of copies of RsbS in the asymmetric unit. Thus, the structure of RsbS was determined by the single-wavelength anomalous dispersion (SAD) method using a crystal of selenomethionine-labeled RsbS. A model with five RsbS monomers (residues 4–119 for chain *A*; residues 5–119 for the other chains) in the asymmetric unit was built and the final model was refined with *R* and *R*_free_ values of 21.0% and 24.1%, respectively (Table 1 ▸). Superposition of the five RsbS monomers in the asymmetric unit revealed that the RsbS proteins in the asymmetric unit have the same conformation (Supplementary Fig. S3). The range of root-mean-square deviations (RMSDs) was 0.4–0.5 Å for 115 C^α^ atoms of the five RsbS monomers.

### 3.2. Overall structure of the RsbS monomer   

The RsbS monomer formed an α/β fold composed of a four-stranded parallel β-sheet (β1–β4) and four α-helices [α1–α4; Figs. 1 ▸(*a*) and 1 ▸(*b*)]. The central β-sheet was sandwiched by the α-helices [Fig. 1 ▸(*b*)]. In addition, a β-strand at the N-terminus (β0) interacted with β1 in the manner of an antiparallel β-sheet. In a structural comparison using *DALI*, RsbS showed high structural similarity to mtSTAS and to *Geobacillus stearothermophilus* SpoIIAB (gsSpoIIAB), both of which are agonists of antisigma factors. mtSTAS and gsSpoIIAB superimposed onto RsbS with RMSD values of 1.6 and 1.9 Å for 112 C^α^ positions, respectively [Fig. 1 ▸(*c*)], indicating that RsbS has the conserved fold of the STAS domain. In addition to the fold of the STAS domain, the monomer of RsbS in the crystal structure was phosphorylated at Ser59, which is associated with the regulation of RsbT binding to the stressosome [Figs. 1 ▸(*a*) and 1 ▸(*b*)]. Additional electron density at Ser59 covering a phosphate was observed and phosphoryl­ation of the peptide including Ser59 was detected by peptide mass fingerprinting (Supplementary Fig. S4). Ser59 was likely to be phosphorylated during protein expression in *E. coli*.

### 3.3. Two interfaces that mediate the packaging of the RsbS crystal   

**Figure 2:**
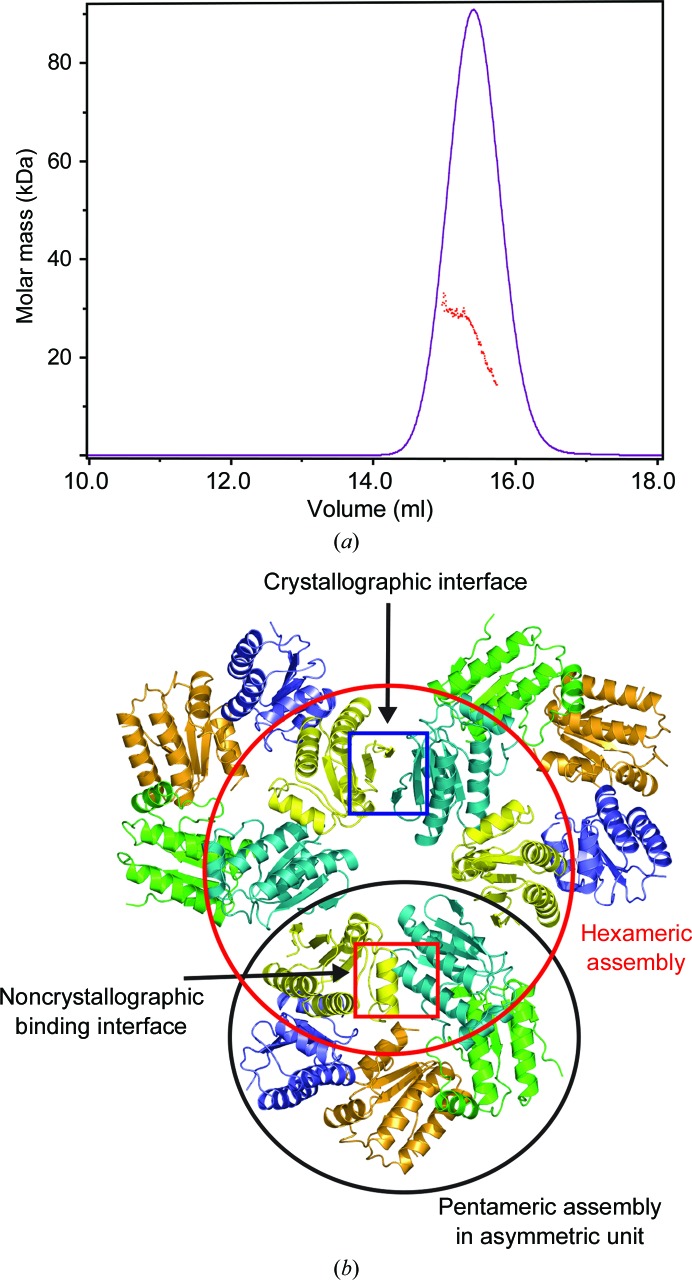
Dimerization of RsbS. (*a*) Oligomeric state of RsbS measured by SEC-MALS. The calculated molecular mass of RsbS was 24.9 kDa, which is close to that of a dimer (the molecular weight of a monomer is 13.3 kDa). (*b*) Binding interfaces in the RsbS crystal. Two dimeric interfaces in the crystal structure of RsbS are shown in the red and blue rectangular boxes. The RsbS hexamer in the red circle is formed by crystallographic and noncrystallographic interfaces, whereas the RsbS pentamer in the black circle is formed by a noncrystallographic interface (the asymmetric unit).

The RsbS crystal contains five monomers in the asymmetric unit (Supplementary Fig. S5*a*). Because the oligomeric state in the asymmetric unit does not always indicate the quaternary structure in solution, the oligomerization state of RsbS in solution was measured by size-exclusion chromatography with multi-angle light scattering [SEC-MALS; Fig. 2 ▸(*a*)]. The molecular size of RsbS in solution was calculated to be a dimer (approximately 24.9 kDa). The RsbS S59A mutant, which restricts phosphorylation, was also calculated to be a dimer, indicating that the phosphorylation on Ser59 does not change the oligomeric state (Supplementary Fig. S6). Thus, the oligomeric state of RsbS in solution does not match the number of RsbS monomers in the asymmetric unit.

**Figure 3:**
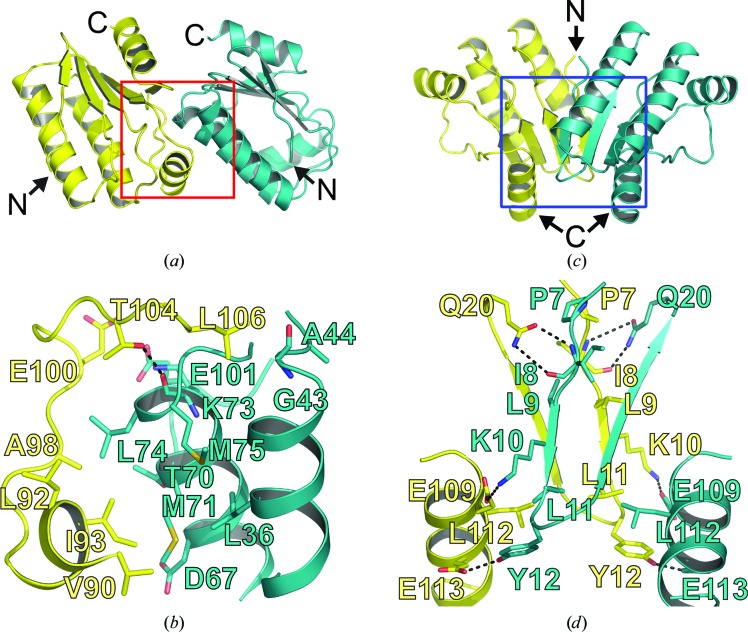
Interactions between two RsbS monomers. (*a*) The dimer structure in an asymmetric unit. The area in the red box indicates the binding interface (interface 1) which mediates the dimerization. The RsbS pentamer in the asymmetric unit is formed by the ‘head-to-tail’ interactions. (*b*) Residues at interface 1 involved in direct contacts. The residues are drawn as stick models and are labeled. (*c*) Dimer structure generated by crystallographic twofold symmetry. The N-termini in the dimeric unit are adjacent to each other. The area in the blue box indicates interface 2. (*d*) The residues involved in the dimerization on interface 2 are drawn as stick models and are labeled. Each monomer in (*a*)–(*d*) is colored differently. The N- and C-termini are labeled N and C, respectively.

Two binding interfaces that mediate crystal packing were observed in the crystal structure of RsbS. Interface 1 was associated with the formation of the asymmetric unit. A pentameric ring in the asymmetric unit was formed by tandem ‘head-to-tail’ interactions of five RsbS monomers [Fig. 2 ▸(*b*)]. The main force for the binding was hydrophobic interactions between residues around α3 and β4 of one RsbS monomer (Val90, Leu92, Ile93, Ala98, Glu100 and Leu106) and residues on α1 and α2 of a neighboring RsbS (Leu36, Gly43, Ala44, Asp67, Thr70, Met71, Leu74 and Met75). In addition, a single hydrogen bond was observed between the side-chain O atom of Thr104 and the backbone O atom of Lys73 [Fig. 3 ▸(*a*) and 3 ▸(*b*)]. A surface area of 663 Å^2^ was buried in the binding interface. Interface 2 mediated the interaction between two RsbS pentamers generated by crystallographic symmetry [Fig. 2 ▸(*b*)]. The RsbS monomers from each pentamer formed a dimer. The dimerization was mainly mediated by residues located at the N- and C-termini. Ionic bonds between Lys10 and Glu109* (where * indicates a residue in a neighboring pentamer), hydrogen bonds (from Tyr12 to Glu113* and from Gln20 to the backbone carbonyl group of Ile8*) and hydrophobic interactions mediated by Pro7, Leu9, Leu11 and Leu112 result in the burial of 721 Å^2^ [Figs. 3 ▸(*c*) and 3 ▸(*d*)].

As explained above, interface 1 was associated with the formation of a pentameric ring, whereas interface 2 mediated the dimerization of RsbS pentamers in the manner of ‘head-to-head’ interactions. The binding area of interface 2 was slightly larger than that of interface 1. Moreover, the dimeric unit of the STAS domain in RsbRA, formed by ‘head-to-head’ interactions, could be connected to an N-terminal globin dimer because the N-termini of the STAS domains were closely packed [Fig. 3 ▸(*c*)]. Thus, the dimer formed by interface 2 seems to be close to a biological dimeric unit of the RsbS domain in the stressosome, which is consistent with the SEC-MALS result.

### 3.4. Superposition of the crystal structure of RsbS onto the stressosome core   

**Figure 4:**
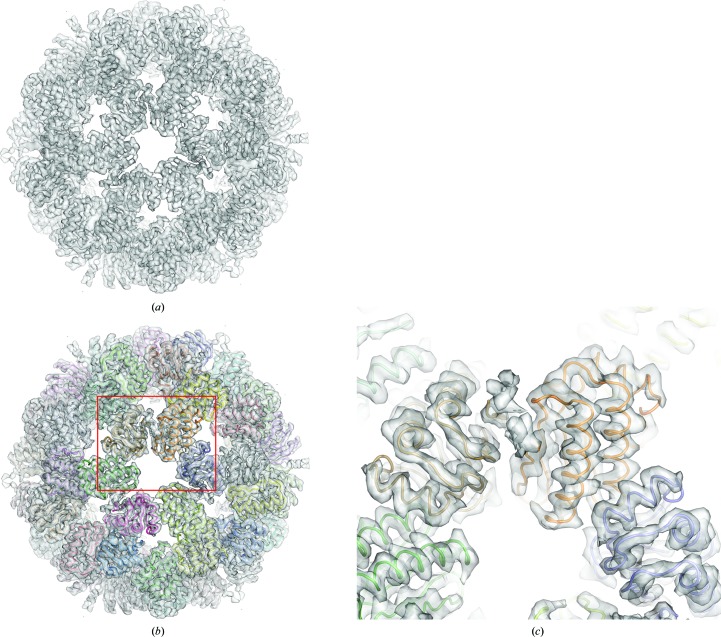
Cryo-EM structure of a stressosome. (*a*) Envelope structure of the RsbRA–RsbS complex calculated at 4.1 Å resolution under an icosahedral symmetric restraint (*I* envelope). (*b*) The RsbS icosahedron superposed onto the *I* envelope. Each RsbS monomer was fitted into the *I* envelope after the crystal structure of the RsbS icosahedron had been superposed. (*c*) Enlargement of the area shown in the red box in (*b*).

A stressosome requires RsbS and at least one RsbR paralog to form a large protein complex (Kim *et al.*, 2004 ▸; Delumeau *et al.*, 2006 ▸; Marles-Wright *et al.*, 2008 ▸). Both RsbR paralogs and RsbS contain STAS domains that directly assemble into a pseudo-icosahedral stressosome core. In the crystal structure of RsbS, the asymmetric unit contained five RsbS monomers, each of which could form a dimer with RsbS from a neighboring pentamer [Fig. 2 ▸(*b*)]. Thus, the centers of three pentamers formed a hexameric structure that comprised three RsbS dimers [Fig. 2 ▸(*b*) and Supplementary Fig. S5(*b*)]. 12 pentamers generated by the crystallographic symmetry of the cubic crystal (space group *I*23) formed the icosahedral ball shape that corresponded to the structure in the unit cell of an RsbS crystal (overall dimensions of 180 × 180 × 180 Å; Fig. 4 ▸), although RsbS itself did not aggregate into a stressosome core in solution [Fig. 2 ▸(*a*)]. It is unclear how RsbS forms an icosahedron in crystal packing. The crystallization condition suggests that the enhanced hydrophobicity from high salt such as 1 *M* NaCl in interface 1 might contribute to the packaging. In SEC experiments, RsbS precipitated in the size-exclusion column using high-salt buffer, indicating that RsbS can aggregate in the presence of high salt but that the stability of RsbS itself may not be sufficient to maintain a large oligomer.

Next, the cryo-EM structure of the RsbRA–RsbS complex was determined for comparison with the crystal structure of the RsbS icosahedron (Supplementary Fig. S1). The RsbRA–RsbS complex was purified by nickel-affinity chromatography with 6×His-thioredoxin-tagged RsbRA and was further purified by SEC after removing the tag. The cryo-EM structure was reconstructed from 42 623 particles selected from cryo-EM images under nonsymmetric *C*1, dihedral *D*2 and icosahedral *I* restraints (*I*, *D*2 and *C*1 envelopes, respectively). The resolutions of the *I*, *D*2 and *C*1 envelopes were evaluated to be 9.1, 7.3 and 4.1 Å, respectively, using the FSC = 0.143 criterion (Supplementary Fig. S2).

When the RsbS icosahedron from the crystal structure was superposed onto the stressosome core of the *I* envelope, it almost fitted into the envelope (Supplementary Fig. S7*a*), and each RsbS monomer was further fitted into the *I* envelope individually (Fig. 4 ▸). The model of the RsbS icosahedron corrected by fitting into the *I* envelope only showed a slightly larger diameter than the crystal structure of the RsbS icosahedron (Supplementary Fig. S7*b*). The RMSD value between the corrected model and the crystal structure of the RsbS icosahedron was 2.6 Å for a total of 6912 C^α^ atoms. Moreover, the backbone of the model of the RsbS icosahedron fitted well into the *I* envelope at the level of secondary structure, although the STAS domains of the stressosome core in the *I* envelope were averaged by icosahedral symmetry (Fig. 4 ▸ and Supplementary Fig. S8). This indicates that the STAS domains of RsbRA and RsbS which constitute the stressosome core harbor a highly conserved fold, and the model of the RsbS icosahedron (thus named the STAS icosahedron) can be used as an atomic model for understanding the stressosome assembly.

### 3.5. The model of stressosome assembly   

The envelope structure of a stressosome calculated under a *D*2 symmetric restraint has previously been reported (Marles-Wright & Lewis, 2008 ▸). The structure suggested that 40 RsbRA and 20 RsbS molecules aggregate to form a stressosome. A total of 20 protrusions that correspond to 20 dimers of the RsbRA N-domain were observed under *D*2 symmetry. Our cryo-EM results showed that the protrusion patterns differ between the *C*1 and *D*2 envelopes (Supplementary Fig. S9). 22 protrusions, which indicate that 44 RsbRA and 16 RsbS monomers are assembled into the stressosome, were observed in the *C*1 envelope, whereas 20 were observed in the *D*2-envelope model; this structure is the same as that reported previously (Marles-Wright *et al.*, 2008 ▸). It is difficult to interpret why the *C*1 envelope and the *D*2 envelope display different protrusion patterns. It is probable that the *D*2 restraint during 3D reconstruction might induce deformation of the pattern. In this case, 3D reconstruction with a *C*1 restraint can minimize the possibility of deformation by symmetric restraints and result in an envelope model that is close to the real structure.

**Figure 5:**
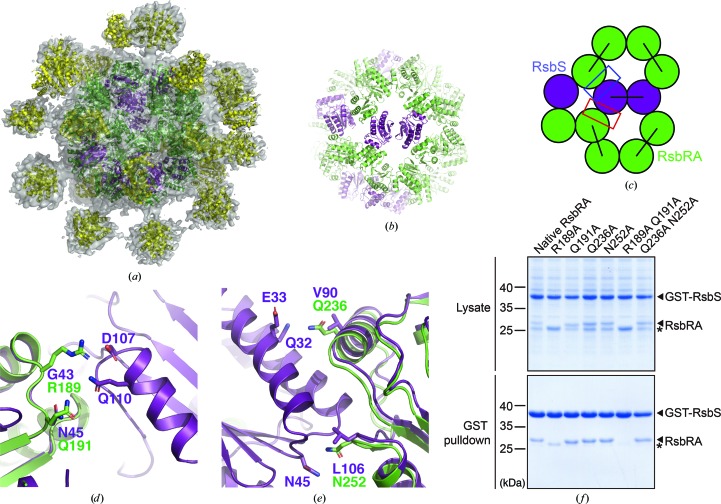
The model of stressosome assembly. (*a*) Envelope structure of the RsbRA–RsbS complex determined under a nonsymmetric restraint at 9.1 Å resolution (*C*1 envelope). The model of the STAS icosahedron and the crystal structure of the RsbRA N-domain (PDB entry 2bnl; Murray *et al.*, 2005 ▸) were superposed onto the icosahedral core and protrusions of the *C*1 envelope, respectively. The position of the RsbRA STAS domains was determined based on the protrusions corresponding to the RsbRA N-domain. The STAS domains of RsbRA and RsbS and the RsbRA N-domain are colored green, purple and yellow, respectively. (*b*, *c*) Ribbon diagram (*b*) and cartoon (*c*) showing the organization of the STAS domains in the stressosome core. (*d*, *e*) Two distinctive binding interfaces between RsbRA and RsbS (interface A and interface B) are indicated by the red and blue boxes in (*c*). The model of the stressosome core was superposed onto the STAS icosahedron which comprises 60 RsbS monomers to show the difference in interaction between RsbS–RsbS and RsbRA–RsbS. Interface A and interface B are shown in (*d*) and (*e*), respectively. RsbRA and RsbS are colored green and purple, respectively. Polar residues of RsbRA at the binding interface that is not conserved between RsbRA and RsbS are drawn as green stick models and labeled. (*f*) GST pulldown of RsbS. Cell lysates (upper panel) that contain GST-RsbS and RsbRA (native or mutants) were prepared and loaded onto GST resin. Proteins bound to the resin were eluted and confirmed by SDS–PAGE (lower panel). The interaction between RsbRA and RsbS was decreased by R189A and R189A/Q191A mutations in RsbRA. * indicates the positions of the R189A and R189A/Q191A mutants of RsbRA on the SDS–PAGE.

In this regard, the position of the RsbRA STAS domain in the stressosome core was identified using the *C*1 envelope. The icosahedral core and 22 protrusions in the *C*1 envelope superposed well with the STAS icosahedron [Fig. 5 ▸(*a*)] and the crystal structure of the RsbRA N-terminal domain, respectively (Supplementary Fig. S10; Murray *et al.*, 2005 ▸). Each protrusion was in the middle of an STAS dimer and was connected to the STAS N-terminus. This indicates that an STAS dimer lying under the protrusion corresponds to that of RsbRA in the stressosome core. The atomic model of the stressosome core was generated based on the superposition (Fig. 5 ▸). The homology model of the RsbRA STAS domain was prepared using the RsbS structure as a template. RsbS dimers in the STAS icosahedron lying under the RsbRA N-domain were replaced with the homology model of the RsbRA STAS domain. Finally, the model of the stressosome core was prepared after energy minimization [Fig. 5 ▸(*b*)].

In the model of the stressosome core, two distinctive binding interfaces between RsbS and the RsbRA STAS domain (interface A and interface B) were observed in the pentameric ring, as shown in the dimeric interface of the RsbS pentameric ring [interface 1; Figs. 3 ▸(*a*) and 3 ▸(*b*)]. The surface areas buried in interfaces A, B and 1 were calculated to be 531 Å^2^ [Fig. 5 ▸(*d*)], 600 Å^2^ [Fig. 5 ▸(*e*)] and 528 Å^2^ [Fig. 3 ▸(*b*)], respectively. The burial of surface area in interface B was increased by 14%, and more hydrophobic interactions were observed compared with those in the STAS icosahedron, whereas that in interface A was not considerably changed.

### 3.6. Ionic and hydrogen bonds between RsbRA and RsbS contribute to stressosome assembly   

Either RsbRA or RsbS forms a dimer and does not aggregate into an icosahedral shape in solution. That is, both RsbRA and RsbS are assembled together to reconstruct a stressosome. Because the fold of the STAS domain is highly conserved in RsbRA and RsbS [Fig. 1 ▸(*a*)], the nonconserved residues lying on the binding interface between RsbRA and RsbS might be critical for stressosome assembly. In this regard, we explored which residues are critical for stressosome assembly, based on the model of the stressosome core. In the sequence alignment between RsbS and the RsbRA STAS domain, Gly43, Val90 and Leu106 of RsbS lying at the binding interface in the STAS icosahedron were aligned with Arg189, Gln236 and Asn252 of RsbRA, respectively [Figs. 1 ▸(*a*), 5 ▸(*d*) and 5 ▸(*e*)]. The charged or polar residues of RsbRA are likely to mediate stronger interactions between RsbS and RsbRA, because the polar residues of RsbS are close to them [Figs. 5 ▸(*d*) and 5 ▸(*e*)]. To investigate whether mutations of the residues can mediate stressosome disassembly, GST (glutathione *S*-transferase) pulldown was performed using RsbRA mutants that disrupt ionic or hydrogen bonds with RsbS in the model of the stressosome core. Six alanine mutants of RsbRA (R189A, Q191A, R189A/Q191A, Q236A, N252A and Q236A/N252A) were prepared to disrupt the ionic or hydrogen bonds at the binding interface between RsbRA and RsbS. All RsbRA mutants and GST-RsbS were overexpressed in *E. coli* and the interaction between RsbS and RsbRA (native or mutant form) was observed using the GST pulldown assay [Fig. 5 ▸(*f*)].

GST-RsbS was co-purified with native RsbRA in the GST pulldown. RsbRA mutations at interface B (Q236A, N252A and Q236A/N252A) did not disrupt the interaction with RsbS, whereas those at interface A affected the interaction [Fig. 5 ▸(*f*)]. In particular, a double mutation of RsbRA at interface A (R189A/Q191A) abolished RsbS binding (Fig. 5 ▸). This indicates that the ionic interaction between Arg189 of RsbRA and Asp107 of RsbS at interface B might be critical for stressosome assembly. Taken together, the model of the stressosome core shows how RsbRA and RsbS assemble into a pseudo-icosahedron and suggests that nonconserved residues of the STAS domains of RsbRA and RsbS mediate the assembly. 

## Supplementary Material

Supplementary Figures S1-S10 and Supplementary Table S1. DOI: 10.1107/S205225251900945X/fq5006sup1.pdf

PDB reference: RsbS, 6jhk

## Figures and Tables

**Table 1:** Data-collection and refinement statistics for the determination of the crystal structure of RsbS

	RsbS	SAD data, peak
Data collection		
Space group	*I*23	*I*23
*a* = *b* = *c* (Å)	179.2	179.8
α = β = γ (°)	90	90
Resolution (Å)	30.0–3.10 (3.15–3.10)	30.0–3.20 (3.26–3.20)
Wavelength (Å)	0.97933	0.97926
Total/unique reflections	103314/17310	155460/16127
Completeness (%)	98.9 (100.0)	99.9 (100.0)
〈*I*/σ(*I*)〉	38.9 (5.2)	41.8 (6.6)
*R*_merge_ (%)	7.2 (54.6)	0.112 (0.565)
Figure of merit		0.356
Refinement		
Resolution	30.0–3.10	
No. of reflections (working/free)	17280/1732	
*R*_work_/*R*_free_ (%)	21.0/24.1	
No. of protein atoms	4437	
*B* factor (Å^2^)	92.7	
RMSD		
Bond lengths (Å)	0.004	
Bond angles (°)	0.717	
Ramachandran plot		
Favored	97.9	
Allowed	1.9	
Disallowed	0.2	

**Table 2:** Cryo-EM data-collection and refinement statistics

Data set	Dihedral symmetric model	Icosahedral symmetric model	Nonsymmetric model
Data collection			
Microscope	Titan Krios G2	Titan Krios G2	Titan Krios G2
Detector	Falcon III DED	Falcon III DED	Falcon III DED
Acceleration voltage (kV)	300	300	300
Mode	Frame mode	Frame mode	Frame mode
Nominal magnification	47000×	47000×	47000×
Defocus range (µm)	1.5–3	1.5–3	1.5–3
Dose per frame (e^−^ Å^−2^)	1	1	1
Exposure time (s)	1.8	1.8	1.8
No. of movie frames	30	30	30
Pixel size (Å)	1.4	1.4	1.4
Movies	1788	1788	1788
Refinement			
Software	*RELION*-2.1	*RELION*-2.1	*RELION*-2.1
Box size (pixel)	330	330	330
Initial No. of particle images	54798	54798	54798
Final No. of particle images	42623	42623	42623
Map resolution (Å) (FSC = 0.143)	7.3	4.1	9.1
Map-sharpening *B* factor (Å^2^)	−303.954	−255.194	−846.868
Symmetry imposed	*D*2	*I*	*C*1
