# Supplementary material for: Structural insights into stressosome assembly
Source: IUCrJ. 2019 Aug 21;6(Pt 5):938–47. doi: 10.1107/S205225251900945X (PMC6760441; doi:10.1107/S205225251900945X)
Supplement: Supplementary file 1 [file m-06-00938-sup1.pdf]

IUCrJ

Volume 6 (2019)

Supporting information for article:

Structural insights into stressosome assembly

Eunju Kwon, Deepak Pathak, Han-ul Kim, Pawan Dahal, Sung Chul Ha, Seung Sik Lee, Hyeongseop Jeong, Dooil Jeoung, Hyeun Wook Chang, Hyun Suk Jung and Dong Young Kim

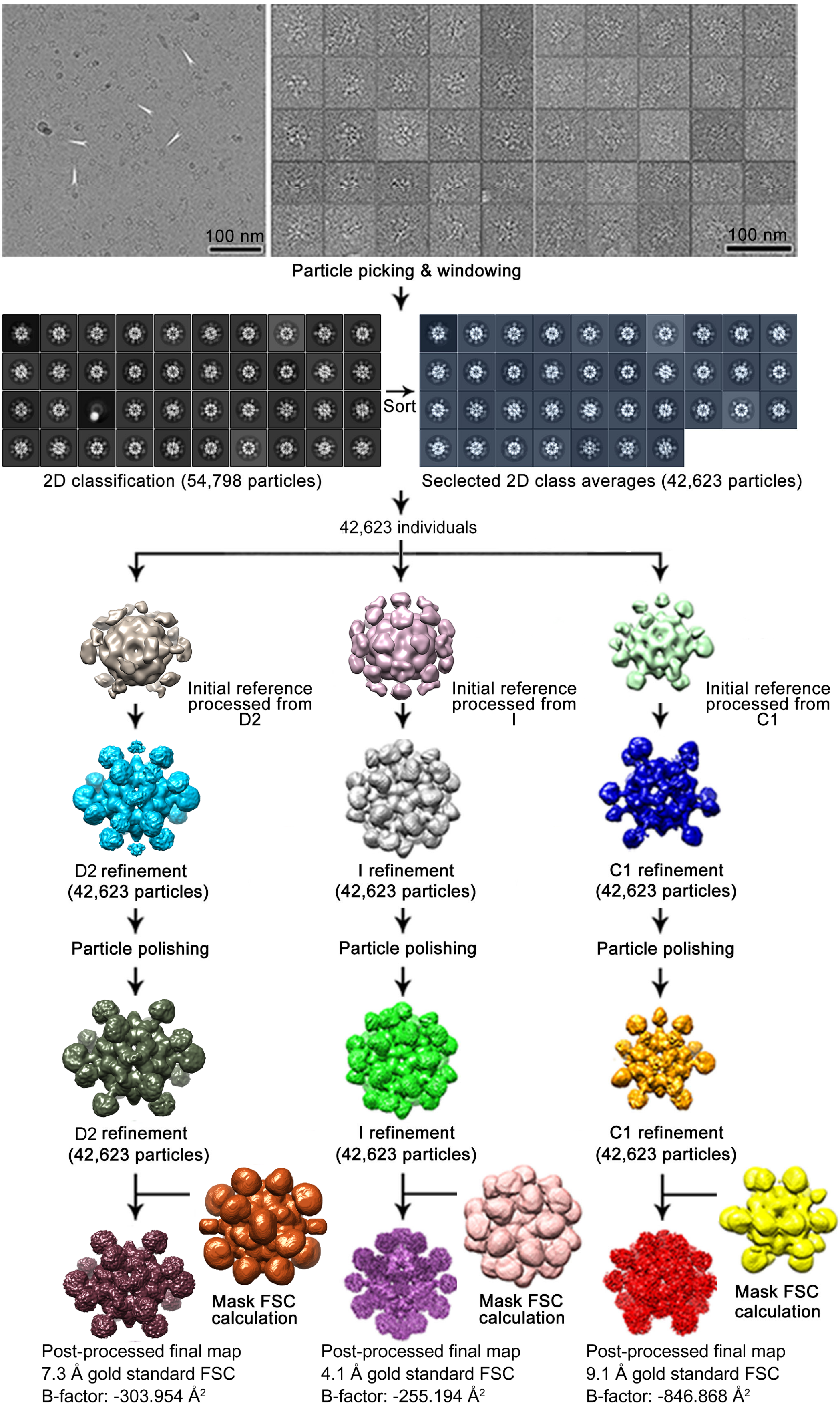
100 nm
100 nm
Particle picking & windowing
Sort
2D classification (54,798 particles)
Seclected 2D class averages (42,623 particles)
42,623 individuals
Initial reference processed from D2
Initial reference processed from I
Initial reference processed from C1
D2 refinement (42,623 particles)
I refinement (42,623 particles)
C1 refinement (42,623 particles)
Particle polishing
Particle polishing
Particle polishing
D2 refinement (42,623 particles)
I refinement (42,623 particles)
C1 refinement (42,623 particles)
Mask FSC calculation
Mask FSC calculation
Mask FSC calculation
Post-processed final map
7.3 Å gold standard FSC
B-factor: -303.954 Å2
Post-processed final map
4.1 Å gold standard FSC
B-factor: -255.194 Å2
Post-processed final map
9.1 Å gold standard FSC
B-factor: -846.868 Å2

Figure S1. Details of Cryo-EM data processing. The workflow that represents 3D reconstruction of RsbRA/RsbS complex. Cryo-EM envelope structures of RsbRA/RsbS complex were refined with dihedral (D2), icosahedral (I), and non-symmetric (C1) restraints.

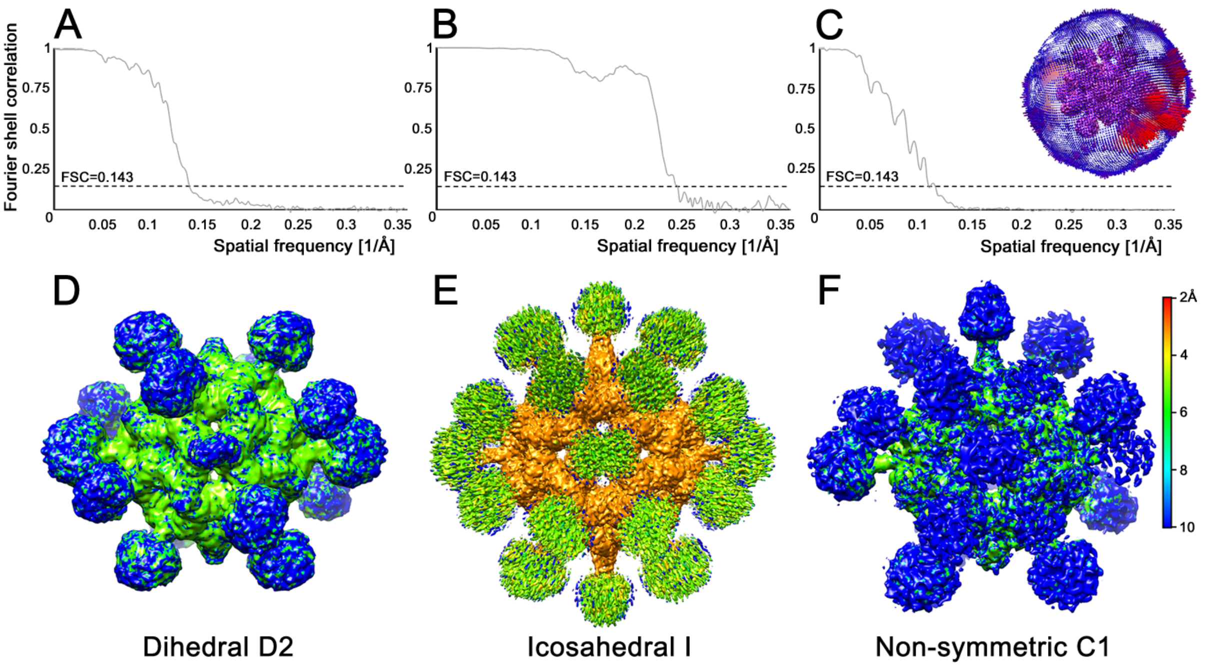

Figure S2. Fourier shell correlation (FSC) and local resolution distributions of Cryo-EM envelope structure. (A-C) FSC curves of D2- (A), I- (B) and C1-envelopes (C) indicates final resolution of 7.3 Å, 4.1 Å, and 9.1 Å, respectively. The resolution was estimated by FSC = 0.143 criterion. The inset image in (C) shows the angular distribution of particles contributing to the final reconstruction of C1-envelope. (D-E) The distribution of local resolution in D2- (D), I- (E), and C1-envelopes (F).

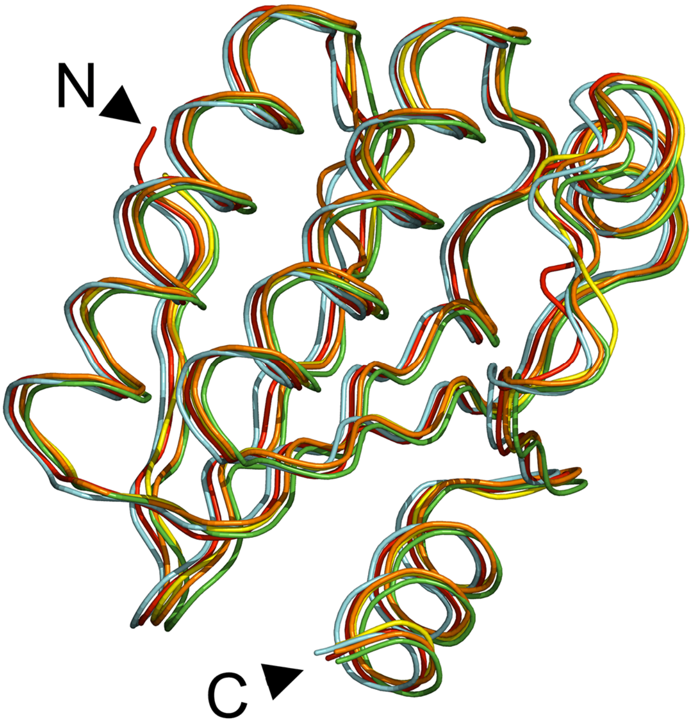

Figure S3. The superposition of five RsbS monomers in an asymmetric unit. Cα trace models of the five RsbS monomers were superposed together at the same orientation as Fig 1C. Each monomer is colored differently. The N- and C-termini are labeled as N and C, respectively.

A

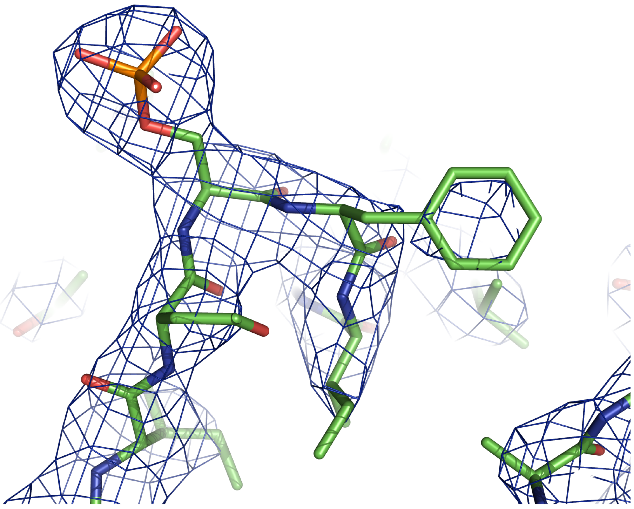

B

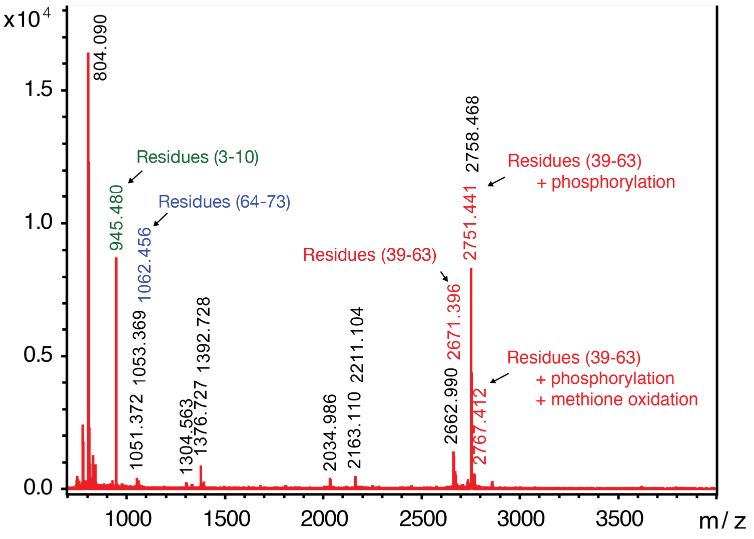

C

```
  1  MRHPKIPILK LYNCLLVSIQ WELDDQTALH FQEDLLNKIY ETGANGVVID
 51  LTSVDMIDSF IAKVLGDVIT MSKLMGAKVV LTGIQPAVAV TLIELGIALE
101  EIETALDLEQ GLETLKRELG E
```

Figure S4. Phosphorylation of S59. (A) Electron density map around S59 of RsbS. RsbS are drawn as a stick model. 2Fo-Fc electron density map is shown at 1.5 σ contour level. (B) Peptide mass fingerprinting (PMF). The peptides from purified RsbS were analyzed by MALDI-TOF and identified by peptide mass fingerprinting. (C) The peptide containing S59 (red) was phosphorylated.

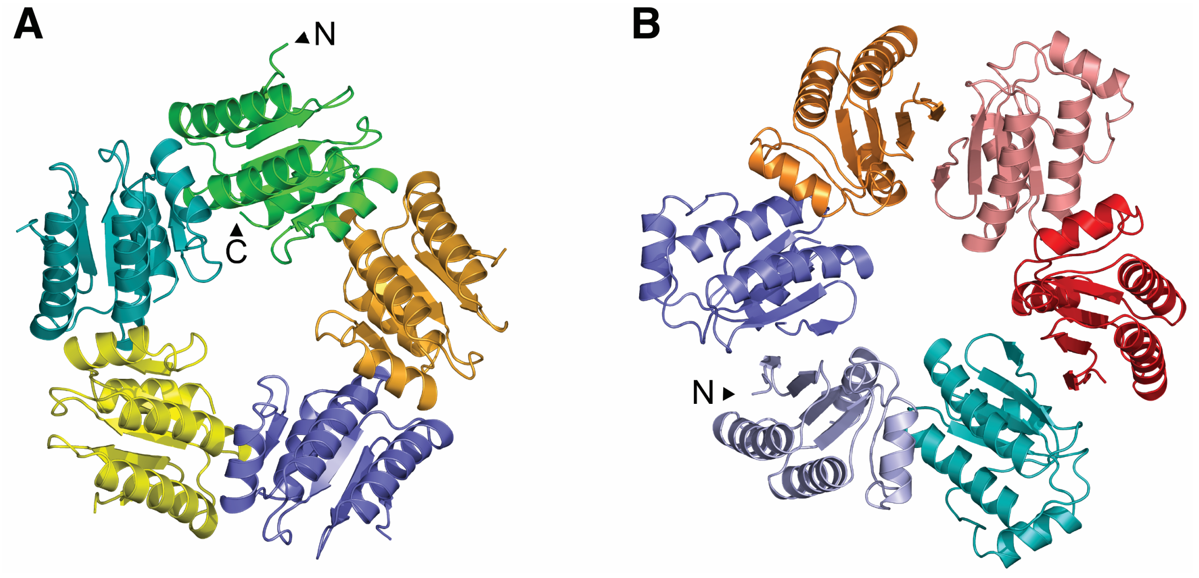

Figure S5. Oligomerization of RsbS shown in the crystal. (A) Ribbon diagram of RsbS pentamer in the asymmetric unit. The pentamer is formed by the “head to tail” interactions of RsbS. Each RsbS monomer is colored differently. (B) RsbS hexamer (trimer of RsbS dimer) generated by crystallographic symmetry.

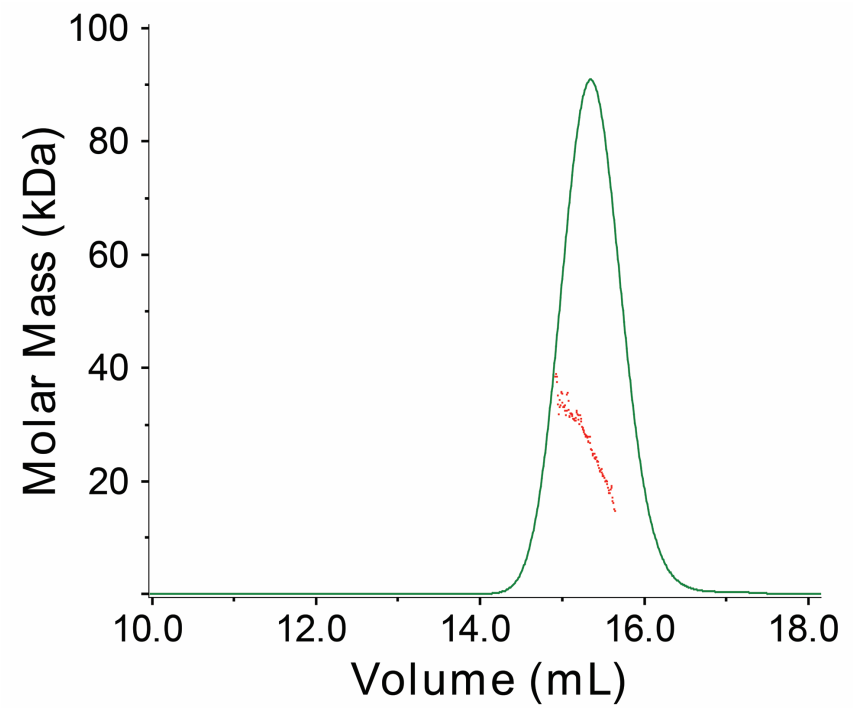

Figure S6. SEC-MALS of RsbS-S59A mutant. The calculated molecular mass of RsbS S59A was 26.8 kDa, which is close to that of a dimer (molecular weight of a monomer: 13.3 kDa).

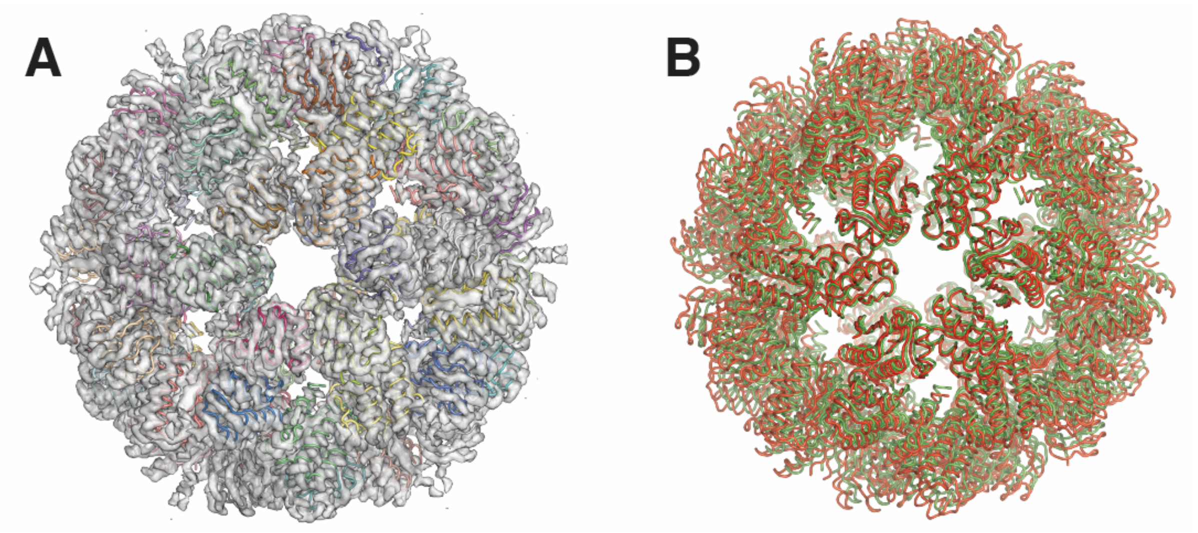

Figure S7. Superposition of the crystal structure of RsbS icosahedron onto the icosahedral envelope. (A) The crystal structure of RsbS icosahedron was drawn as ribbon diagram and was fitted into the cryo-EM envelope of RsbRA/RsbS complex determined with icosahedral symmetric restraint at 4.1 Å resolution. (B) Comparison of RsbS crystal structure and STAS icosahedron. To generate STAS icosahedron, each RsbS monomer from the RsbS crystal structure was fitted into the I-envelope, individually. RsbS crystal structure and STAS icosahedron were colored green and red, respectively.

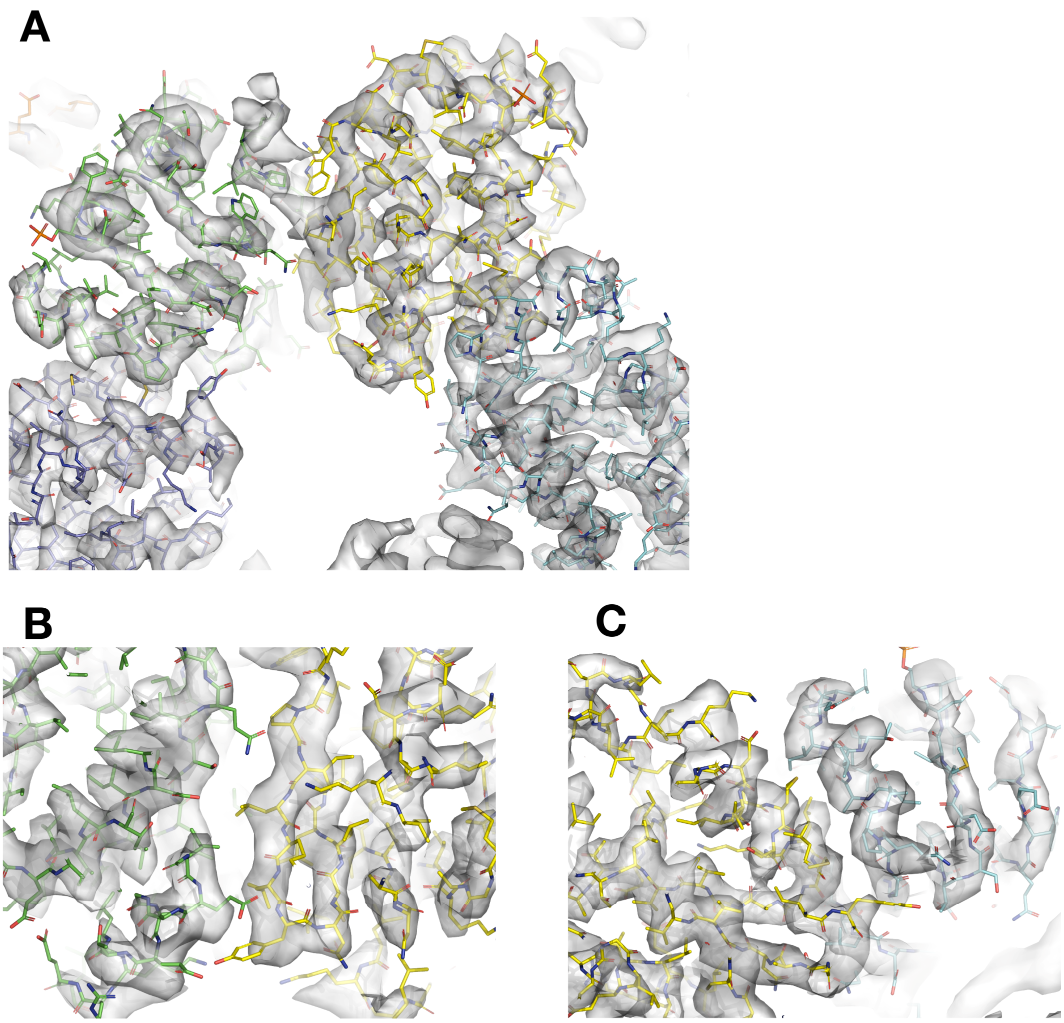

Figure S8. Icosahedral envelope of the RsbRA/RsbS complex (A-C) Envelope structure superposed with crystal structure of RsbS. The crystal structure of RsbS icosahedron was superposed on the icosahedral envelope calculated at 4.1 Å resolution. Individual RsbS monomer was then fitted into the envelope. RsbS monomers from the crystal structure are drawn as a stick model and differently colored. (B, C) Structure of binding interfaces.

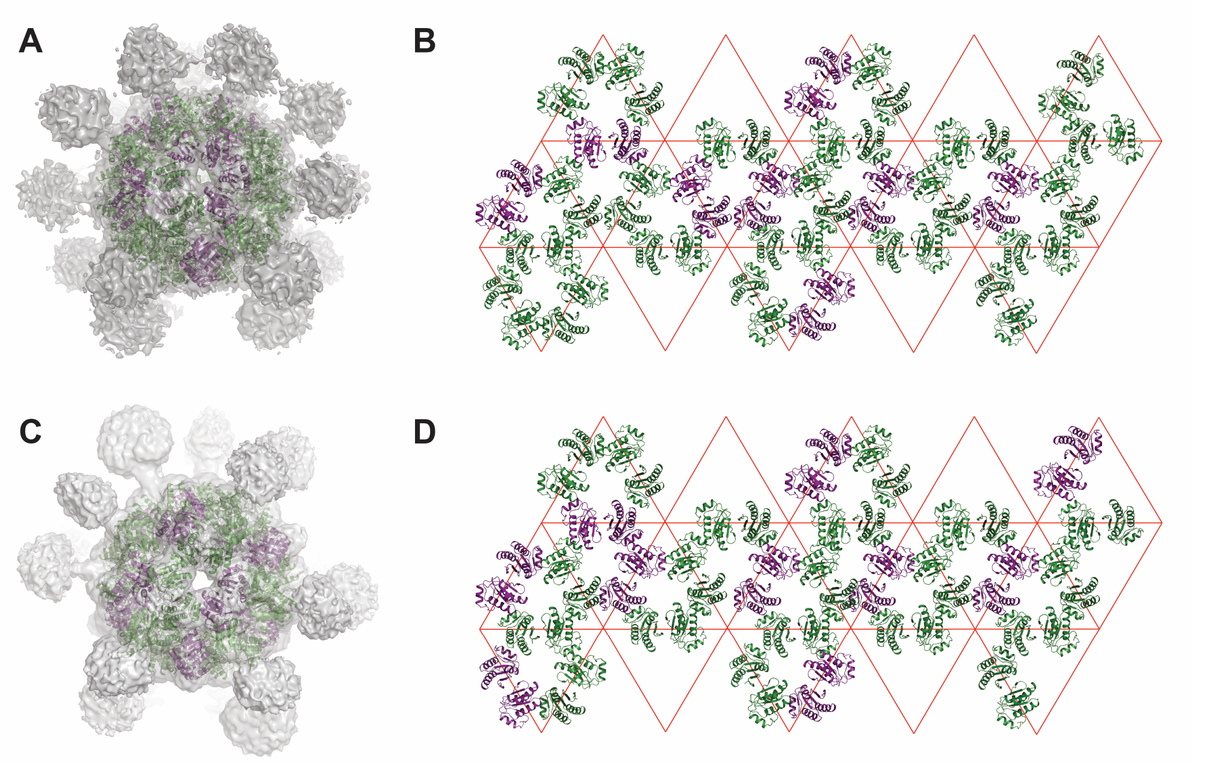

Figure S9. Cryo-EM structure of a stressosome (A) Envelope structure of RsbRA/RsbS complex calculated at 9.1 Å resolution without symmetric restraint (C1-envelope). Ribbon diagram of RsbS icosahedron was superposed onto the C1 envelope. The positions of RsbRA-STAS domain and RsbS in stressosome core were distinguished by green and purple, respectively. (B) The flattened icosahedron that shows the arrangement of RsbRA and RsbS in the C1 envelope. (C) Envelope structure of RsbRA/RsbS complex calculated at 7.3 Å resolution under D2 symmetric restraint (D2-envelope). (D) The flattened icosahedron that shows the arrangement of RsbRA and RsbS in the D2 envelope.

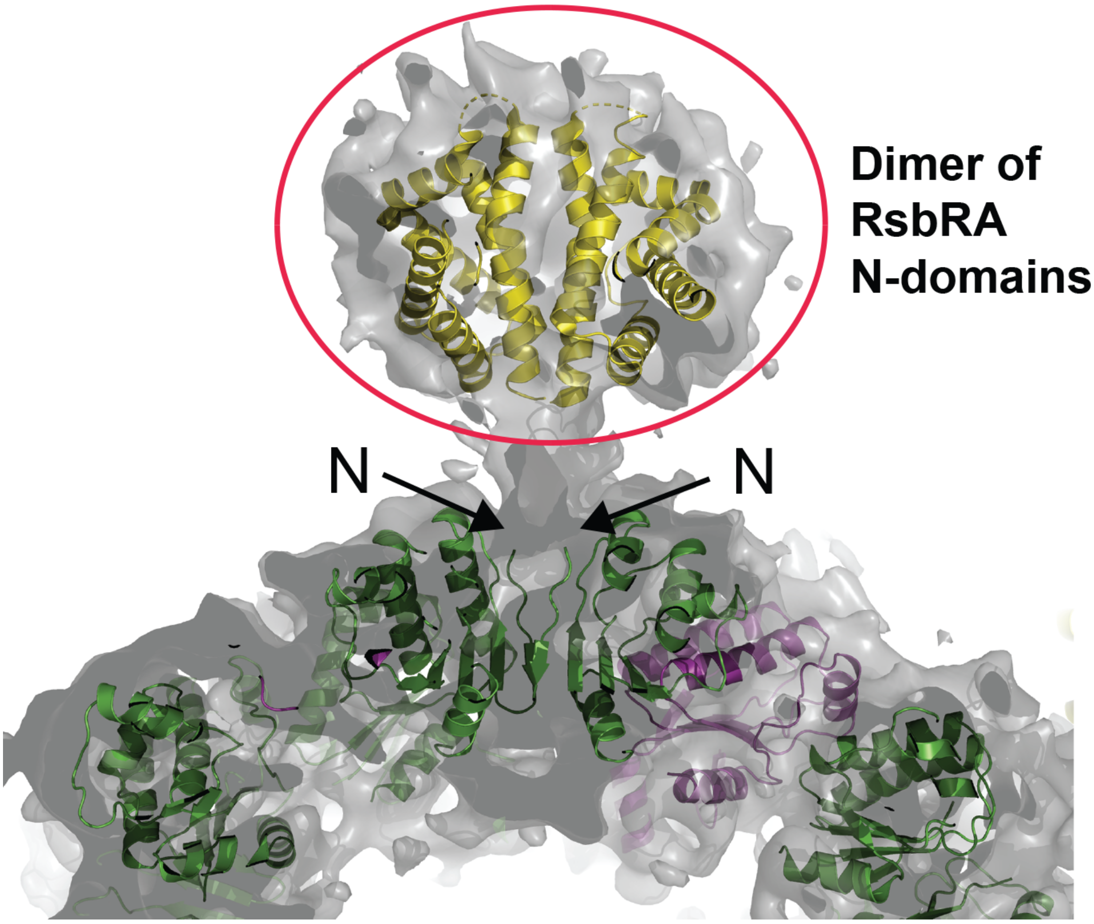

Figure S10. Superposition of domain models on C1 envelope of stressosome. The STAS domains of RsbRA and RsbS and RsbRA N-domain are colored green, purple, and yellow, respectively.

Supplementary Table S1. Genes and primers used

Genes	UniProt accession number (www.uniprot.org)
RsbRA	P42409
RsbS	P42410
RsbT	P42411

Primers	DNA sequence (5' → 3')
BsRsbS_F	ACTGGATCCATGAGACATCCGAAAATCCCGATCTT
BsRsbS_R	AATGAATTCCTATTCCCCCAATTCCCGCTTCAAT
BsRsbRA_F	ACTGGATCCATGATGTCGAACCAGACTGTATACCA
BsRsbRA_R	ATGATAAGCTTTTATTCCCCCAATGAAACGATTTTT
BsRsbRA_R189A_ F	GTCAAACACGCGTCCCAAGTCGTGCTGATTGACATTACA
BsRsbRA_R189A_R	GACTTGGGACGCGTGTTTGACGACGCCATTAAGAAGGTT
BsRsbRA_Q191A_F	CACCGTTCCGCGGTCGTGCTGATTGACATTACAGGTGTC
BsRsbRA_Q191A_R	CAGCACGACCGCGGAACGGTGTTTGACGACGCCATTAAG
BsRsbRA_Q236A_F	GAAATTGCTGCGACAATCGTCAACCTGGGGATTGATTTA
BsRsbRA_Q236A_R	GACGATTGTCGCAGCAATTTCCGGACGGATTCCGGCCAG
BsRsbRA_N252A_F	ATAACTAAGGCGACCCTGCAAAAAGGAATTCAAACAGCA
BsRsbRA_N252A_R	TTGCAGGGTCGCCTTAGTTATAACTTGGGATAAATCAAT
BsRsbRA_189Ato189A191A_F	CACGCGTCCGCGGTCGTGCTGATTGACATTACAGGTGTC
BsRsbRA_189Ato189A191A_R	CAGCACGACCGCGGACGCGTGTTTGACGACGCCATTAAG
BsRsbT_F	TGACATTACATATGAACGACCAATCCTGTGTAAGAAT
BsRsbT_R	AACTATCACTCGAGCTACCGAAGCCATTTGATGGCTTGT
